# Multichannel Surface EMG Decomposition Based on Measurement Correlation and LMMSE

**DOI:** 10.1155/2018/2347589

**Published:** 2018-06-28

**Authors:** Yong Ning, Yuming Zhao, Akbarjon Juraboev, Ping Tan, Jin Ding, Jinbao He

**Affiliations:** ^1^School of Automation and Electrical Engineering, Zhejiang University of Science and Technology, Hangzhou 310023, China; ^2^School of Computing, University of Portsmouth, Portsmouth PO1 3HE, UK; ^3^China Coal Research Institute, Beijing 100013, China; ^4^Ningbo University of Technology, Ningbo 315211, China

## Abstract

A method based on measurement correlation (MC) and linear minimum mean square error (LMMSE) for multichannel surface electromyography (sEMG) signal decomposition was developed in this study. This MC-LMMSE method gradually and iteratively increases the correlation between an optimized vector and a reconstructed matrix that is correlated with the measurement matrix. The performance of the proposed MC-LMMSE method was evaluated with both simulated and experimental sEMG signals. Simulation results show that the MC-LMMSE method can successfully reconstruct up to 53 innervation pulse trains with a true positive rate greater than 95%. The performance of the MC-LMMSE method was also evaluated using experimental sEMG signals collected with a 64-channel electrode array from the first dorsal interosseous muscles of three subjects at different contraction levels. A maximum of 16 motor units were successfully extracted from these multichannel experimental sEMG signals. The performance of the MC-LMMSE method was further evaluated with multichannel experimental sEMG data by using the “two sources” method. The large population of common MUs extracted from the two independent subgroups of sEMG signals demonstrates the reliability of the MC-LMMSE method in multichannel sEMG decomposition.

## 1. Introduction

Electromyographic (EMG) signals are comprised of action potentials produced by the muscle fibers contained in different motor units (MUs) [1]. It is of great importance for physiological investigation and clinical diagnosis to decompose EMG signals into their constituent motor unit action potential (MUAP) trains. EMG signal decomposing will lead to a better understanding of the properties of MU control and reveal the MUAP changes due to muscle fiber denervation/reinnervation [2]. It will also aid in the examination of neuromuscular diseases (e.g., amyotrophic lateral sclerosis) and the process of evaluating the degree of dysfunction found in upper motoneuron diseases such as Parkinson's disease [3], cerebral palsy [4], hemiparetic stroke [5], and other disorders [6, 7]. Furthermore, EMG decomposition can facilitate the study of the interpulse interval (IPI) variability [8], recruitment strategies [9], myoelectrical manifestations of fatigue [10], and short-term MU synchronization [11].

EMG signals can be detected by introducing a fine wire or needle sensor into the muscle tissue or by placing sensors on the surface of the skin. In the course of studying these EMG signals, it has been found that the surface detection of EMG provides several advantages over wire or needle detection. For example, surface electrodes can be used quickly and easily, without causing discomfort for the subject or requiring medical supervision [12], and measurements can be performed with a high degree of repeatability. More importantly, surface EMG (sEMG) is also able to obtain global information about muscle activities and consequently records a vast amount of information [12]. This makes it more convenient for studying neuromuscular control mechanisms than the invasive methods, which offer less information about global muscle activities and are more difficult to utilize.

Over the past few decades, great strides have been made in decomposing indwelling EMG signals [13–15]. However surface EMG decomposition remains a difficult task. There is routinely a high level of action potential overlapping and cancellation within sEMG signals. The volume conduction effect for propagating action potentials is also enhanced in surface recordings due to the relatively large distance between electrodes and sources [12]. In addition, there exists the spatial integrating effect caused by surface electrodes. Hence, the differences in surface action potential shapes from different MUs are not as distinguishable as with intramuscular recordings [12]. Together, all of these factors make sEMG decomposition an extremely difficult task, especially at high force levels.

Various approaches for sEMG decomposition have been proposed over the past years in both sEMG recording and processing [16–21]. In particular, the design of surface electrode arrays comprised of a number of tiny electrode probes with a small interelectrode distance promises to increase the motor unit discrimination capacity by reducing MUAP superimposition while providing spatial information across the muscle. The extraction of a single MU from sEMG has also become a feasible task at very low force levels with appropriate signal processing methods such as two-dimensional template matching [20]. Recent developments in sEMG decomposition have further allowed for the extraction of a number of simultaneously firing MUs at relatively high force levels. Nawab et al. [17] developed a remarkable sEMG decomposition technique using a specially designed 5-pin Laplace electrode array in conjunction with a knowledge-based artificial intelligence framework. Holobar and Zazula proposed the convolution kernel compensation (CKC) method [18] and the gradient CKC approach (GCKC) [19] to decompose multichannel sEMG signals recorded with high density electrode arrays. It has been demonstrated that the GCKC method holds the promise of high efficiency and a strong antinoise performance in sEMG decomposition [19], but it has a strict requirement for the length of the EMG signals for its iterative process to converge. It has become easier to a certain extent for decomposing multichannel SEMG signals which are originally difficult to process since the CKC method was introduced into the field of SEMG decomposition. Other multichannel signal processing methods have also been tested with high density sEMG decomposition, including traditional template matching, independent component analysis, higher order cumulants, and correlation measurement, but most of these methods have been limited to relatively low muscle contraction levels.

In view of the existing facts, it is hard to decompose complex superposition sEMG signals. Moreover, the decomposing procedure is also a bit cumbersome. For example, it usually needs multiple steps to build a correlation vector between the IPT and the measurements in the past. While in this article, it only needs an iterative procedure to form the correlation vector. A method based on measurement correlation and linear minimum mean square error (MC-LMMSE) was developed in this study to decompose multichannel sEMG signals. The MC-LMMSE method is firstly used to reconstruct a matrix correlated with the measurement matrix. Then, it gradually and iteratively increases the correlation between an optimized vector and a reconstructed matrix until a satisfactory innervation pulse train (IPT) is obtained. The performance of the MC-LMMSE method was assessed with both simulated and experimental sEMG signals. The results demonstrated that the MC-LMMSE method can successfully extract more MUs and reconstruct IPTs with a higher true positive rate (TPR) than the GCKC method, even from complex superposition signals.

## 2. Materials and Methods

### 2.1. Data Model

Multichannel sEMG signals can be modelled as a linear-time-invariant multi-input-multi-output system [22] if the muscle contraction is maintained at a constant force level. This system can be represented by the matrix form as follows [18]:

(1) $$\begin{array}{c} X\left(n\right)=G\bar{s}\left(n\right)+W\left(n\right), \end{array}$$

where *X*(*n*)=[*x*_1_(*n*),…,*x*_*M*_(*n*)]^*T*^ is the *M* measurements, *x*_*j*_(*n*) is the *n*th sample of the *j*th measurement, *W*(*n*)=[*w*_1_(*n*),…,*w*_*M*_(*n*)]^*T*^ stands for a vector of zero-mean white noise, *G* denotes a mixing matrix which consists of all of the channel responses *g*_*ij*_=[*g*_*ij*_(0),…, *g*_*ij*_(*Q* − 1)] (the *j*th source in sEMG signals appearing in the *i*th measurement) of *Q* samples, and $\bar{s}\left(n\right)$ is an extended form of the *N* sources *s*(*n*)=[*s*_1_(*n*),…,*s*_*N*_(*n*)]^*T*^ can be described as $\bar{s}\left(n\right)={\left[s_{1}\left(n\right),s_{1}\left(n-1\right),\ldots ,s_{1}\left(N-Q+1\right),\ldots ,s_{N}\left(n\right),s_{N}\left(n-1\right),\ldots ,s_{N}\left(n-Q+1\right)\right]}^{T}$.

### 2.2. Method of LMMSE

Given a vector form **X**=[*x*(0), *x*(1),…, *x*(*N*−1)] whose probability density function (PDF) is unknown, the linear estimator of a variable **θ** related to the **X** statistics can be written as follows:

(2) $$\begin{array}{c} \hat{\theta }=\overset{N-1}{\underset{n=0}{\sum }}a_{n}x\left(n\right)+a_{N}. \end{array}$$

Choose the weighting coefficients *a*_*n*_'s to minimize the Bayesian mean square error (MSE):

(3) $$\begin{array}{c} \text{Bmse }\left(\hat{\theta }\right)=E_{X\theta }\left\{{\left(\theta -\hat{\theta }\right)}^{2}\right\}, \end{array}$$

where the resultant estimator $\text{Bmse }\left(\hat{\theta }\right)$ is termed the linear minimum mean square error (LMMSE) estimator [23]. Substituting (2) in (3), then it becomes

(4) $$\begin{array}{c} \text{Bmse }\left(\hat{\theta }\right)=E\left\{{\left[\theta -\overset{N-1}{\underset{n=0}{\sum }}a_{n}x\left(n\right)-a_{N}\right]}^{2}\right\}. \end{array}$$

Differentiating and setting this equal to zero,

(5) $$\begin{array}{c} \frac{\partial \;\mathrm{Bmse}\;\left(\hat{\theta }\right)}{\partial a_{N}}=-2E\left\{{\left[\theta -\overset{N-1}{\underset{n=0}{\sum }}a_{n}x\left(n\right)-a_{N}\right]}^{2}\right\}=0. \end{array}$$

Then, it produces

(6) $$\begin{array}{c} a_{N}=E\left(\theta \right)-\overset{N-1}{\underset{n=0}{\sum }}a_{n}E\left[x\left(n\right)\right]. \end{array}$$

Substituting (6) in (4), then it becomes

(7) $$\begin{array}{c} \text{Bmse }\left(\hat{\theta }\right)=E\left\{{\left[\overset{N-1}{\underset{n=0}{\sum }}a_{n}\left[x\left(n\right)-E\left(x\left(n\right)\right)\right]-\left(\theta -E\left(\theta \right)\right)\right]}^{2}\right\}. \end{array}$$

Let **a**=[*a*_0_, *a*_1_,…, *a*_*N*−1_], and it has

(8) $$\begin{array}{c} \text{Bmse }\left(\hat{\theta }\right)=E\left\{{\left[a^{T}\left(X-E\left(X\right)\right)-\left(\theta -E\left(\theta \right)\right)\right]}^{2}\right\}\\ =E\left\{a^{T}\left(X-E\left(X\right)\right){\left(X-E\left(X\right)\right)}^{T}a\right\}-E\left\{a^{T}\left(X-E\left(X\right)\right)\left(\theta -E\left(\theta \right)\right)\right\}\\ \;-E\left\{\left(\theta -E\left(\theta \right)\right){\left(X-E\left(X\right)\right)}^{T}a\right\}+E\left\{{\left(\theta -E\left(\theta \right)\right)}^{2}\right\}\\ =a^{T}C_{XX}a-a^{T}C_{X\theta }-C_{\theta X}a+C_{\theta \theta }. \end{array}$$

Because *C*_*θX*_=*C*_*Xθ*_^*T*^, it has

(9) $$\begin{array}{c} \text{Bmse }\left(\hat{\theta }\right)=a^{T}C_{XX}a-2a^{T}C_{X\theta }+C_{\theta \theta }. \end{array}$$

Equation (9) can be maximized by taking the gradient:

(10) $$\begin{array}{c} \frac{\partial \text{ Bmse }\left(\hat{\theta }\right)}{\partial a}=2C_{XX}a-2C_{X\theta }, \end{array}$$

and setting it to zero, which results in

(11) $$\begin{array}{c} a=C_{XX}^{-1}C_{X\theta }. \end{array}$$

Substituting (6) and (11) into (2) produces

(12) $$\begin{array}{c} \hat{\theta }=E\left(\theta \right)+C_{X\theta }C_{XX}^{-1}\left(X-E\left(X\right)\right). \end{array}$$

If the means of **θ** and *X* are zero, then

(13) $$\begin{array}{c} \hat{\theta }=C_{X\theta }C_{XX}^{-1}X. \end{array}$$

For multichannel sEMG signals, $\hat{\theta }$ is the innervation pulse train (IPT) that needs to be estimated, *X* is the measured multichannel sEMG signal, and *C*_*Xθ*_ is a parameter that needs to be calculated. It has been pointed out in [18] that all firing times of MU need to be known in advance to calculate *C*_*Xθ*_, which can be written as

(14) $$\begin{array}{c} C_{X\theta }=\frac{1}{\mathrm{card}\;\left(\psi_{j}\right)}\sum \bar{X}\left(\psi_{j}\right), \end{array}$$

where set *ψ*_*j*_ contains all firing times of the same MU and $\bar{X}$ is the extended form of the measured signal *X*. The LMMSE estimator can be obtained after substituting (14) in (13). In fact, it is very hard to know MU firing times beforehand. In view of this, a method that is able to identify complete or most of firing time of MU was proposed; therefore, we can achieve the results or approach results of LMMSE.

### 2.3. Measurement Matrix Autocorrelation

Multiplied by a 1 × M vector *v* from both sides, (1) becomes

(15) $$\begin{array}{c} vX\left(n\right)=vG\bar{s}\left(n\right)+vW\left(n\right). \end{array}$$

The *i*th IPT in $\bar{s}\left(n\right)$ can be calculated with (16) if *vG*=[0, 0,…,1, 0,…,0]_1×NQ_ (suppose the value of the (*i*−1)*Q*+*r*+1th element is 1, and all other values are 0), and the noise term *W*(*n*) is negligible.

(16) $$\begin{array}{c} s_{i}\left(n-r\right)\approx vX\left(n\right),\;0\leq r\leq Q-1. \end{array}$$

In practice, it is difficult and even impossible to find such a vector *v* if the mixing matrix *G* is unknown, but *s*_*i*_(*n* − *r*) can still be satisfactorily reconstructed as long as one of the elements in *v* is far greater than others.

The similarity *S*_AB_ between vectors A and B can be evaluated as follows [24]:

(17) $$\begin{array}{c} S_{\text{AB}}=\frac{\text{Inner}\left[\text{A},\text{ B}\right]}{\left\|\text{A}\right\|\cdot \left\|\text{B}\right\|}, \end{array}$$

where Inner[·] denotes the inner product and ‖·‖ denotes the norm. The shapes of the MUAPs generated by the same MU should have a certain degree of similarity when the isometric muscle contraction is held at a constant force. Therefore, the inner product of two vectors which are associated with different time instants fired by the same MU, should be relatively large. This property provides the possibility to estimate the IPTs of MUs with the following equation:

(18) $$\begin{array}{c} P_{j}\left(n_{i}\right)=vX\left(n_{i}\right)=\text{Inner}\left[v,\;X\left(n_{i}\right)\right],\;n_{i}=1,2,\ldots ,N_{s}, \end{array}$$

where *P*_*j*_(*n*_*i*_) is the value of the estimated innervation pulse train *P*_*j*_(*n*) at the sample time *n*_*i*_, *N*_s_ denotes the number of sample times in each channel, and *v* is a 1 × M vector. If *v* has a strong correlation with the measurement vectors associated with the time instants fired by a particular MU, the firing pattern of this MU will be easily observed in *P*_*j*_(*n*). The vector *v*, then, increases the values in *P*_*j*_(*n*) at time instants when this MU is firing and decreases other values at time instants when it is not. The following average form [18] can be used as *v* to achieve such a purpose:

(19) $$\begin{array}{c} v=\frac{1}{N_{v}}\sum X\left(\varphi_{nv}\right), \end{array}$$

where *φ*_*nv*_={*n*_*v*1_, *n*_*v*2_,…, *n*_*vn*_} denotes the time instants fired by the particular MU, *X*(*φ*_*nv*_) is the series of measurement vectors associated with *φ*_*nv*_, and *N*_*v*_ is the number of elements in *φ*_*nv*_. An ideal *v* will have a stronger correlation with all the measurement vectors contained in *X*(*φ*_*nv*_) and, in this case, due to the average result, *P*_*j*_(*φ*_*nv*_) should be larger than other values in *P*_*j*_(*n*) and can be easily observed. It is difficult, however, to find a satisfactory vector *v*, as the firing pattern of any MU is unknown in practice. As a result, it is necessary to develop an advanced approach to better estimate *v* in order to successfully reconstruct the IPTs.

### 2.4. Measurement Correlation Based on LMMSE (MC-LMMSE)

An iterative algorithm based on LMMSE is developed in the proposed MC-LMMSE method to gradually optimize the vector *f* in order to achieve a better IPT reconstruction. Assuming *Y*(*n*)_*M*×*Ns*_ is a matrix which has a certain column correlation with *X*(*n*), then the IPT estimation equation can be rewritten as

(20) $$\begin{array}{c} s_{i}\left(n-r\right)\approx f_{1\times M}Y{\left(n\right)}_{M\times Ns},\;0\leq r\leq Q-1, \end{array}$$

where the vector *f* plays the same role as the aforementioned vector *v*. Replace *X*(*n*) with *Y*(*n*) in (19) and the vector *f* can be rewritten as

(21) $$\begin{array}{c} f=\frac{1}{N_{v}}\sum \left[Y\left(\varphi_{nv}\right)\right]. \end{array}$$

In this article, the matrix *Y*(*n*) in the MC-LMMSE method is reconstructed from unitary matrices obtained from the singular value decomposition (SVD) of the measurement matrix *X*(*n*) (see Step 1 below). Other matrices can also be selected. The high column correlation of the matrix *Y*(*n*) helps the MC-LMMSE increase the values of *s*_*i*_(*n*−*r*) in (20) at the time instants fired by the same MU. Hence, the influence of noise on its IPT estimation results is significantly suppressed.

**Figure 1:**
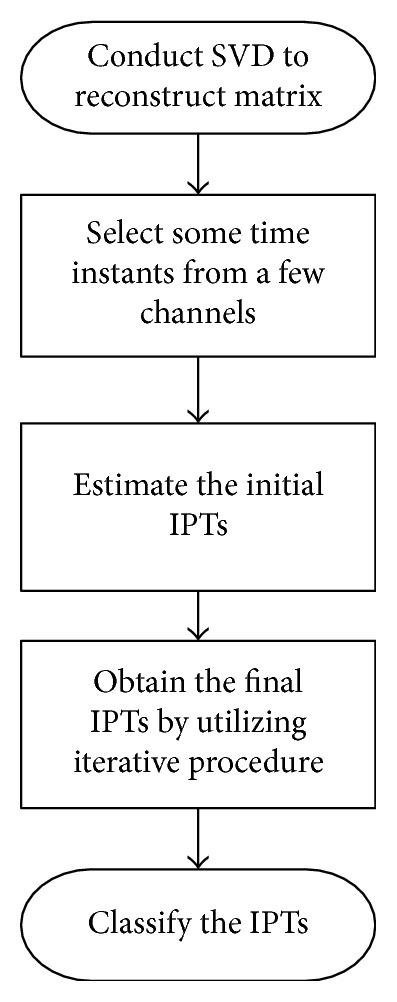
Schematic outline of the proposed MC-LMMSE algorithm.

An initial vector *f* will first be formed from any time instants fired by an MU. The MC-LMMSE method will then be implemented by following the steps listed below to make *f* approximate the ideal vector in (21) and to reconstruct future IPTs with high accuracy. The schematic outline of the MC-LMMSE is shown in Figure 1.

1. Decompose the matrix *X*^*T*^  (*n*)  into  *X*^*T*^  (*n*)=UDV^*T*^ using SVD, where *T* denotes the transpose, and estimate the matrix *Y*(*n*)_*M*×*Ns*_=[*U*_*Ns*×*M*_*V*_*M*×*M*_^*T*^]^*T*^.
2. Randomly select sEMG signals from a few channels and denote each channel signal by *X*_*j*_(*n*); calculate the Teager energy operator [25] of *X*_*j*_(*n*), *ξ*_*nj*_=*X*_*j*_(*n*)^2^ − *X*_*j*_(*n* − 1)*X*_*j*_(*n*+1), and set a threshold (thre); identify all the time instants in *ϕ*_*nj*_ which satisfy *ξ*_*nj*_ > thre to form *φ*_*nj*_={*n*_*j*1_, *n*_*j*2_,…, *n*_*jx*_}.
3. Choose *f*_0_=*Y*(*n*_*jx*_)^*T*^, *n*_*jx*_ ∈ *φ*_*nj*_, and then estimate an IPT *P*_*jx*_(*n*)=*f*_0_*Y*_(*n*)_=*Y*_(*njx*)_^*T*^*Y*_(*n*)_ from each time instant in *φ*_*nj*_ according to (20).
4. Identify *d*_*k*_ (the subscript *k* denotes the *k*th iteration) time instants, *φ*_*nx*_={*n*_*x*1_, *n*_*x*2_,…, *n*_*xd*_*k*__} corresponding to the highest peaks for each initial IPT *P*_*jx*_(*n*), where *d*_*k*_=A · B^*k*^+C · *k* (A and C are constants greater than or equal to zero, where in most instances 1 ≤ B ≤ 3), and then replace *f*_0_ with *f*=(1/*N*_*x*_)∑*Y*(*φ*_*nx*_). A new IPT *P*_*jx*_(*n*) will be obtained by substituting *f* into (20). The vector *f* will be gradually improved by repeating this iterative process until *d*_*k*_ > *N*_*p*_ (*N*_*p*_ is a rough estimate number of firing times in each IPT) at which point the final IPT will be obtained.
5. Classify all the IPTs into groups for each specific MU.

Note that after substituting *Y*(*n*) in Step (1) into (20), it is similar to CKC method [18]. Both of them are correlation method in essence. However, it is helpful for simplifying the decomposition expression by using (20) and understanding the distinguishing feature of these correlation methods to decompose sEMG.

The MC-LMMSE and classic CKC have some similarities, which include that (1) they directly estimate IPTs from measurement matrix without involving calculation of unknown mixing matrix *G*, (2) they all need to select some vectors of measurement corresponding to discharged time instants. However, there are also differences between them that lead to different results (see the following section of results). In MC-LMMSE method, a new way to reconstruct matrix correlated with the measurement matrix was proposed (20). Then, sEMG signals can be decomposed by using the reconstructed matrix. In this article, the SVD method was used to reconstruct the correlation matrix. The measurement matrix itself can also be directly used as the correlation matrix (see the previous section of measurement matrix autocorrelation). In addition, other effective ones such as the measurement matrix transformed by FastICA [26] can also be used as the correlation matrix. Hence, it may further obtain better results if the correlation matrix is properly selected in future. Another difference comparing with CKC is the utilization of iterative technique in Step (4) which can achieve more precise IPTs, and more number of MU firing time is an improved iteration method of CKC. Because it can find more number of time instants *φ*_*nv*_ discharged by one MU in the process of gradually and iteratively calculating vector *f* in (21) in terms of the characteristics of SEMG and the algorithm. Therefore, the quality of *f* can be improved a lot when comparing with classic CKC and GCKC methods.

### 2.5. Simulated Signals

#### 2.5.1. Simulated Signals Generated by Random Mixing Matrices

Ten sources were assumed and the IPTs *s*_*i*_(*n*)=∑_*k*=1_^200^*δ*(*n* − 100 · *k*+*S*_*i*_(*k*)) were randomly generated in the simulation with a mean IPI of 100 samples. The lengths of the IPTs were set to 20,000 samples where *S*_*i*_(*k*), *k*=1, 2,…, 200, was uniformly distributed over the interval [−10, 10]. The zero-mean mixing matrix *G* was also randomly generated with a length *g*_*ij*_ of 10 samples. The number of measurements was set to 25 and the number of delayed repetitions of each original measurement was set to 9. Therefore, the number of extended IPTs was increased to 190 with 250 measurements. Gaussian zero-mean noises were added to each signal with different signal-to-noise ratios (SNRs) of −10 dB, −5 dB, 0 dB, 5 dB, and 10 dB. The measurement matrix autocorrelation method did not need to reconstruct the matrix *Y*(*n*) in Step 1, while *Y*(*n*_*jx*_) in Step 3 and *Y*(*φ*_*nx*_) in Step 4 were replaced by *X*(*n*_*jx*_) and *X*(*φ*_*nx*_), respectively. The number of channels and threshold value in Step 1 were set to 10 and 0.45 MA_*nj*_ (MA_*nj*_ denotes the maximum absolute value of *ξ*_*nj*_ in the Step 2), respectively, when the MC-LMMSE method was implemented. The number of iterations to estimate $\hat{c}t_{j}x$ [19] and the number of main decomposition loops were set to 40 and 500, respectively, when the method of GCKC was implemented. The scalar function *f*(*t*)=(1/3)*t*^3^ was taken in (9) from [19]. An IPT was selected as real when its TPR was greater than 75%.

#### 2.5.2. Simulated Signals Generated by Gaussian Function [27]

The extracellular single fiber action potential (SFAP) was depicted by the sum of three basic Gaussian functions [28].

(22) $$\begin{array}{c} \phi \left(t\right)=\overset{3}{\underset{i=1}{\sum }}U_{i}e^{-{\left(\left(t-C_{i}\right)/\left(V_{i}\right)\right)}^{2}}, \end{array}$$

where *t* is time, *U*_*i*_ is the amplitude factor, *V*_*i*_ is the bandwidth, and *C*_*i*_ is the position of the center of the peak. With this equation, one may approximate a particular triphasic action potential waveform with considerable accuracy by adjusting *U*_*i*_ and *V*_*i*_. Each fiber is assumed to be parallel to the skin surface, so the shape of the SFAP detected by the electrodes is considered to be a function of the physiological parameters, such as the fiber location within a 3-dimensional Cartesian coordinate system, and the muscle fiber conduction velocity. *U*_*i*_ and *V*_*i*_ in (22) were depicted as

(23) $$\begin{array}{c} U_{i}=f_{1}\left(x,\;y,\;z,\text{ cv}\right), \end{array}$$

(24) $$\begin{array}{c} V_{i}=f_{2}\left(x,\;y,\;z,\text{ cv}\right), \end{array}$$

where *y* stands for the vertical fiber depth below the surface of the skin, *z* represents the center position along the fiber in the *z-x* plane, *x* is the fiber center position in the *z-x* plane perpendicular to the *z* direction, and cv is the conduction velocity of muscle fiber. The MUAP shapes detected by different electrodes were depicted as the summation of the SFAP shapes contained in the MUs. The MUAP trains were then generated by the convolution of the MUAP shapes with their corresponding firing times. Finally, the composite sEMG signals were modelled as linear summations of the MUAP trains. The characteristics of the MUAP, such as the amplitude distribution, shape, and duration, were determined by the morphological properties of the active muscle fibers contained within corresponding MUs. The SEMG signals can be simulated with considerable similarity by adjusting the parameters of Gaussian functions according to the characteristics of real SEMG signals. In this article, the SEMG signals are just roughly simulated. However, it can still demonstrate the basic characteristics of SEMG.

**Figure 2:**
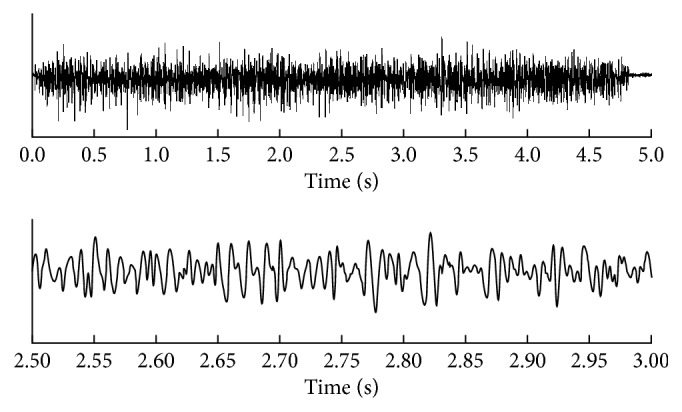
Simulated signals generated by a Gaussian function. The top trace represents one channel of the simulated synthetic signal generated by a Gaussian function with SNR = 20 dB, while the second is an expanded segment (0.5 s) of the raw signal. The average firing rate of all MUs was 20 ± 5 Hz for the 60 MUs that were activated.

The depths of the centers for all measured MUs were uniformly distributed from 1 mm to 6 mm. A random number of fibers (uniformly distributed between 30 mm and 70 mm) were assumed in active MUs. All semifiber lengths were set to 50 mm, and the tendon and endplate positions of the fibers were uniformly distributed in the range of ±5 mm. The conduction velocities of active MUs were set to 4.0 m/s, the firing rates of the MUs were normally distributed with the mean, and standard deviation of 20 ± 5 Hz and 60 active MUs were assumed in total. The starting times of MUs were chosen from 10 ms to 200 ms. A 16 × 16 electrode-array grid with a 3 mm interelectrode distance in both directions was employed for recording the sEMG signals. This grid center was placed at the center of the muscle and the signals were sampled with frequency of 2,000 Hz. The numbers of fibers, position of the active MUs, and discharging patterns were all randomly generated. The signals were also corrupted by additive Gaussian zero-mean noise with SNR of 20 dB as shown in Figure 2. The number of delayed repetitions of each original measurement was set to 9 [18, 19].

### 2.6. Experimental Signals

The experimental sEMG signals were collected from the first dorsal interosseous (FDI) muscles of three adult subjects. The procedures were approved by the Institutional Review Board of Northwestern University (Chicago, USA), and all three subjects gave their written consent before the experiment. Subjects were seated upright in a mobile Biodex chair (Biodex, Shirley, NY). A standard 6 degrees-of-freedom load cell (ATI Inc, Apex, NC) setup was used along with standard procedures for minimizing spurious force contributions from unrecorded muscles as described in [29] to accurately record the isometric contraction force of the FDI muscle during index finger abduction. SEMG signals were recorded from the FDI muscle using a flexible 2-dimensional 64-channel surface electrode array (8 × 8 array with the electrode probe diameter of 1.2 mm, and the center-to-center probe distance of 4 mm) (TMS International BV, The Netherlands) [30]. The skin of the tested muscle was carefully prepared and the electrode array was attached to the FDI muscle with a double adhesive sticker and further secured with medical tapes [29]. The maximum voluntary contraction (MVC) was first measured. Each subject was then asked to generate an isometric contraction force of the FDI muscle at the different contraction levels of 2 N, 4 N, 6 N, and 8 N. Multiple trials were performed with one force level being recorded for each trial. The subject was asked to maintain the force as stable as possible for up to 15 s. A Refa amplifier (TMS International BV, The Netherlands) was used to record sEMG signals. The signals were sampled at 2 kHz with a bandpass filter set at 10–500 Hz. The number of delayed repetitions of each original measurement was set to 9 [18].

### 2.7. Validation

For simulated signals, the parameter TPR and MR defined in (25) and (26) are used to further validate the accuracy of sEMG signal decomposition algorithm, and defined in (26):

(25) $$\begin{array}{c} \text{TPR}=\frac{\text{TP}}{\text{TP}+\text{FN}}, \end{array}$$

(26) $$\begin{array}{c} \text{MR}=\frac{\text{TP}}{\text{TP}+\text{FN}}, \end{array}$$

where TP is the number of correctly identified firing times of pulses in the reconstructed IPT, FP is the number of misplaced discharges, and FN stands for the number of unidentified firing times of pulses in the IPT. For the simulated signals generated by the Gaussian function, the firing time tolerance was set to ±1 sample. Therefore, each identified firing time was considered as true if it was detected within ±0.5 ms (sampling frequency of 2,000 samples/s) from its actual position along the signal. The value defined in (25) was averaged over 10 trials for all identified IPTs. For simulated signals generated by random mixing matrices, the time tolerance was set to 0. The value defined in (25) in this case was also averaged over 10 trials for all identified IPTs.

For experimental signals, to validate the accuracy of MC-LMMSE algorithm, the “two-source” technique, in which all 64 channels of the electrode array were divided into two independent groups with equal number of channels, was used as an alternative to using intramuscular EMG together with surface EMG [16, 17]. The coincident rate of the firing times of the MUs, which are decomposed from both channel groups using the MC-LMMSE algorithm, were calculated, and a high coincident rate was taken to suggest a favourable performance of the algorithm.

## 3. Results and Discussion

### 3.1. Simulated Signals

#### 3.1.1. Tests on Signals Generated by Random Mixing Matrices

Ten trials were conducted to test the performance of the proposed MC-LMMSE method in decomposing the sEMG signals simulated by random mixing matrices and the results were averaged over the 10 trials. The number of reconstructed IPTs, corresponding TPR and MR achieved by the measurement matrix autocorrelation, GCKC and the MC-LMMSE method at different SNRs are presented in Table 1. Results show that the measurement matrix autocorrelation method could not completely reconstruct the IPTs even with a high SNR of 10 dB. The GCKC method only reconstructed an average of 5 IPTs when SNR was set to −10 dB. The MC-LMMSE method reconstructed all the 10 IPTs successfully with the high TPRs at all tested SNR levels (−10 dB to 10 dB) and the TPR maintained over 92% even in severely noisy environments (SNR = −10 dB). Results demonstrate that the MC-LMMSE method offers superior performance to the measurement matrix autocorrelation and GCKC methods of sEMG decomposition. In addition, a parameter called pulse-to-noise-ratio (PNR) [31] was also utilized to evaluate the performance of MC-LMMSE method. The average PNR was 12.37 dB and infinite, respectively, when SNR was set at −10 dB and greater than 0 dB.

#### 3.1.2. Tests on Signals Generated by Gaussian Function

**Figure 3:**
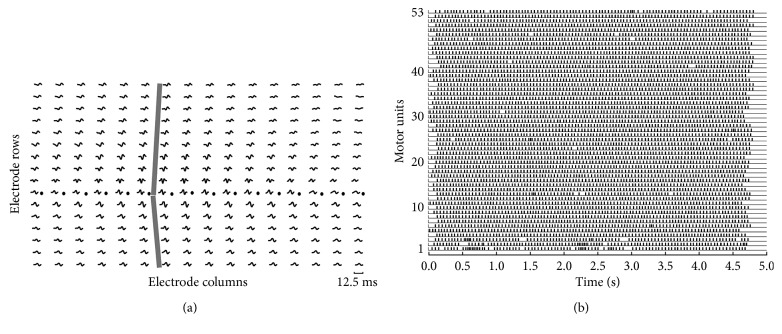
MUAP templates and MU discharge patterns from simulated signals generated by Gaussian functions. (a) Multichannel MUAP templates estimated by the spike-triggered averaging of the simulated sEMG. The locations of the innervation zones (black circles) and the propagation of MUAPs (grey lines) are indicated. (b) MU discharge patterns are identified from the multichannel simulated sEMG signals.

**Figure 4:**
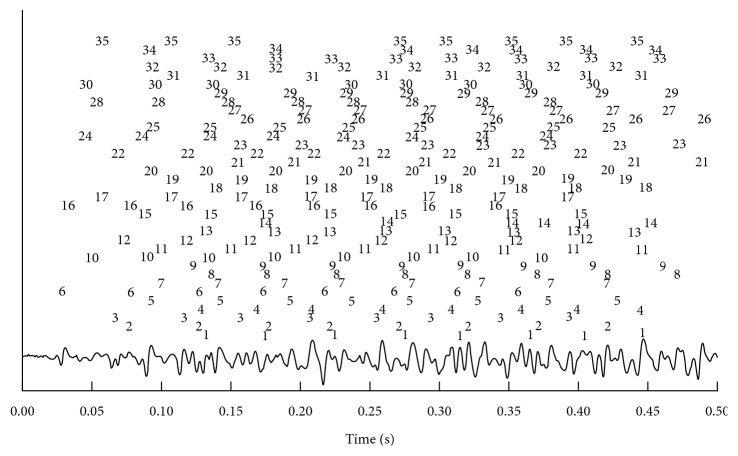
A decomposition example of simulated signals generated by Gaussian functions from one channel. The firing times of each extracted MU are indicated by an assigned label at top of the signal.

The GCKC and MC-LMMSE methods were employed to decompose the sEMG signals generated by a Gaussian function. On average, 26 IPTs were reconstructed by the GCKC method with a TPR of 92.67% and MR of 4.26%; while 53 IPTs were reconstructed by the MC-LMMSE method with a TPR of 97.89% and MR of 1.93% (Figures 3 and 4). The average PNR of MC-LMMSE was 27.39 dB.  Figure 3(a) shows the MUAP shapes of one MU, detected by the 16 × 16 electrode array, which were estimated using the spike-triggered averaging method [32]. The innervation zone of the MU and the propagation of MUAPs can also be clearly observed.

Figure 3(b) shows the 53 IPTs reconstructed from the signals. The firing times of each extracted MU are indicated by an assigned label at top of the signal in Figure 4. Thirty-five MUs can be correctly identified from this channel and the challenge caused by overlapped action potentials appears to be solved by the proposed MC-LMMSE method. The parameters used in the MC-LMMSE and GCKC methods for this test are the same as those used in Test 1, except that the number of main decomposition loops in the GCKC method was set to 5,000.

### 3.2. Tests on Experimental Signals

**Figure 5:**
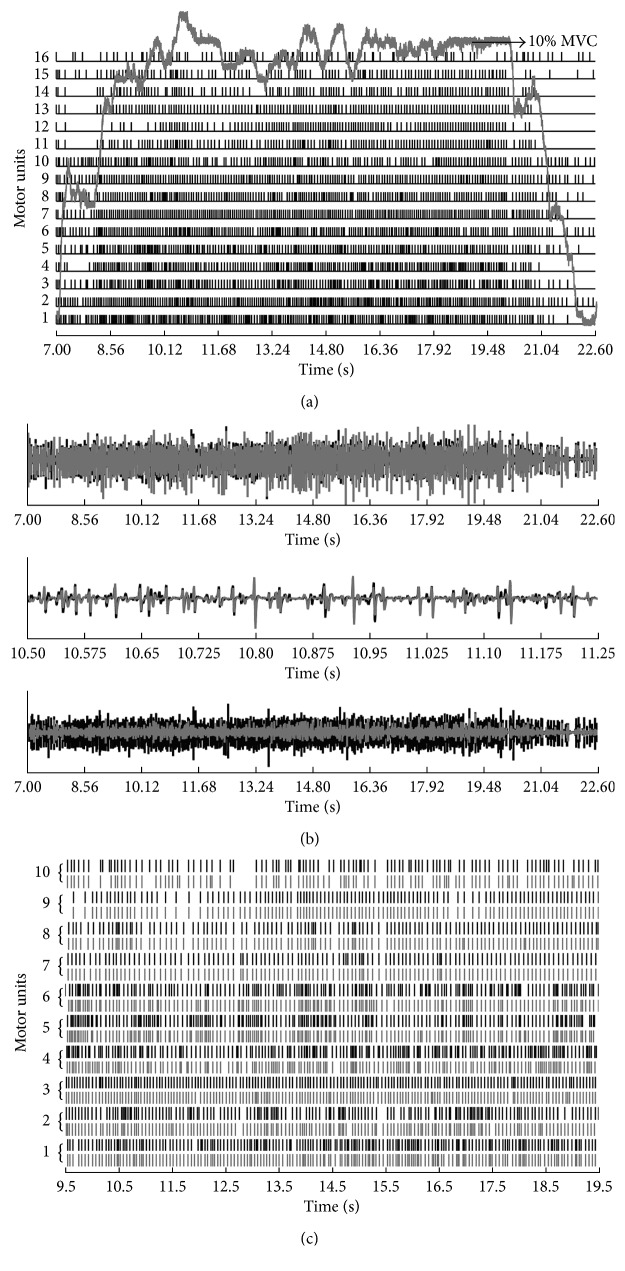
Results obtained from first dorsal interosseous (FDI) muscle. (a) MU discharge patterns with the force profile identified from the FDI muscle during an isometric constant force contraction at 10% MVC (2 N (Subject A). Each vertical line indicates a MU discharge at a given time instant. ((b) Top panel) the sum of identified MUAP trains (grey lines) compared to the raw sEMG signal (black lines) in one selected channel from the first dorsal interosseous (FDI) muscles during an isometric constant force contraction at 10% MVC (Subject A). ((b) Middle panel) an expended view of the top panel. ((b) Bottom panel) the residual (grey lines) compared to the raw sEMG (black lines) after the subtraction of the reconstructed MUAP trains. (c) MU firing patterns identified from Group 1 (black lines) and Group 2 (grey lines). All 64 channel signals were divided into 2 independent groups, with the even numbered columns selected as one group and the odd numbered columns as the other group.

Figure 5(a) shows the force profile and the 16 IPTs identified from the sEMG signals of the FDI muscles by using the MC-LMMSE method. These sEMG recordings were taken during an isometric constant force contraction at 10% of the maximum voluntary contraction (MVC).

**Figure 6:**
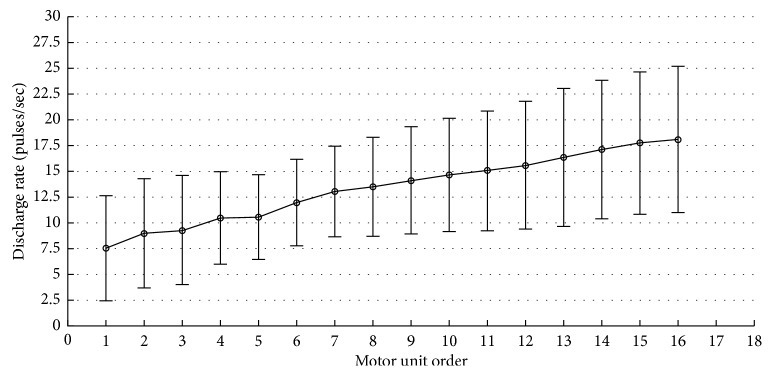
The mean and standard deviation of discharge rates of the extracted 16 MUs from first dorsal interosseous (FDI) muscle during an isometric constant force contraction at 10% MVC (2 N).

It can be seen that the firing rate of MUs changes with the fluctuation of the contraction force; Figure 5(b) compares the summation of the identified MUAP trains and their residuals respective to the original sEMG signals, where the signal-to-interference ratio (SIR) [33] between the sum of identified MUAP trains and raw sEMG signal was 59.73%.  Figure 6 shows the mean and standard deviation of discharge rates of the extracted 16 MUs from FDI muscles. It can been seen from the figure that the average discharge rates of these extracted 16 MUs range from 7.55 ± 5.1 to 18.1 ± 7.1 pulses/second. Different MUs correspond to different average discharge rate patterns, which are monotonically increasing. Considering the individual differences of the physiological characteristics [34], these values may differ slightly from the previous reported results; however, overall they are similar [34, 35].

The results achieved by the GCKC and MC-LMMSE methods are shown in Table 2. It can be seen that the MC-LMMSE method extracted more MUs than the GCKC method, especially in the cases of high force contraction. The parameters used in the MC-LMMSE and GCKC methods in this test are the same as in Test 1. The performance of the MC-LMMSE method with the experimental electrode array sEMG was further investigated by using the “two sources” method. All of the 64 channel signals recorded at different contraction force levels were divided into 2 independent groups, each with 32 channels. SEMG signals recorded from channels with even column numbers were selected to form Group 1, while signals recorded from channels with odd column numbers were selected to form Group 2. The proposed MC-LMMSE method was applied to each of the groups for sEMG decomposition, and the numbers of MUs extracted from all the channel signals, signals in Group 1 and signals in Group 2, were compared (Table 2 and Figure 5(c)). It can be seen that, overall, the number of extracted MUs decreases as the number of EMG channels decreases. This trend becomes more remarkable in cases where a higher force of contraction was applied. It can also be seen that results achieved from the two independent groups share over 84% of the commonly extracted MUs and show over 90% of the same firing times for the common MUs.

## 4. Discussion

One important concept to decompose high density array signals like SEMG is proposed in this article. There are two important steps for decomposing signals which lead to its superior performance compared to other decomposition methods. One is the appropriate selection of the matrix which is correlated to the measurement matrix; the other one is the estimation of the reconstructed IPTs with the iterative optimization process presented in Step 4. Both steps are critical in achieving favourable decomposition results. In fact, ${\bar{X}}^{T}\left(n\right)C_{\bar{XX}}^{-1}$ in [18] can also be considered as a matrix correlated with the measurement matrix. In addition to the mentioned correlated matrix in this article, other matrices have also been found that can decompose sEMG signals. The decomposition results are likely to improve in near future. However, like other decomposition methods, the MC-LMMSE method also has some limitations. For example, there ought to be at least thousands of samples in sEMG signals, otherwise if the length of signals is too short, it will be difficult to obtain satisfactory results. It can be seen from the results of the simulation data that this MC-LMMSE method requires a larger number of detected electrodes to get better results. But only 64 electrodes were used to record the real sEMG signals in this article. Hence, if hundreds of electrodes could be employed to record the real signals, there is hope that a larger number of MUs could be extracted, and the allowable force of muscle contraction could also become larger.

The matrix *Y*(*n*) in the MC-LMMSE method is constructed with a high level of column correlation from the unitary matrices obtained using the SVD of the measurement matrix. This high column correlation is able to help the MC-LMMSE suppress the influence of noise, as the correlation between vector *f* and the other vectors from *Y*(*n*) associated with the firing times of the same MU is further enhanced by the iterative optimization procedure in Step 4. (In fact, the results obtained by MC-LMMSE can better approach the LMMSE estimator when compared with CKC which is derived from LMMSE estimator. Please refer to [18] for further understanding why MC-LMMSE method can get such results.) Therefore, both the employment of a SVD of the measurement matrix and the iterative optimization procedure in the MC-LMMSE contribute to the improvement of the decomposition performance when compared to the other methods tested in this paper (Figure 3, Tables 1 and 2). The time instants in each iteration step corresponding to the highest peaks in *p*_*jx*_(*n*) are usually the firing times of a particular MU, making it possible to employ such an optimizing approach to improve the vector *f*. Both the decomposition method presented in this study and CKC method are based on high density surface EMG recordings; however, the MC-LMMSE method employs a different approach for IPT estimation. It differs from CKC that (21) was adopted in the proposed MC-LMMSE algorithm to gradually optimize the vector *f* and give it a stronger correlation with the different vectors from matrix *Y*(*n*) associated with the firing time instants of a particular MU. Instead, Equation (20) is utilized in the MC-LMMSE method to estimate the IPTs, where *Y*(*n*) is reconstructed by the unitary matrices obtained through the SVD of *X*(*n*), and the vector *f* is obtained by an iterative optimization procedure. The final IPTs can then be obtained by substituting *f* into (20). CKC and GCKC are the two typical sEMG signal decomposition methods. Moreover, GCKC can get better results compared with CKC [19], hence we chose GCKC method as a comparison here. The following relevant published articles have little improvement in performance and many of them are related applications for decomposition. It should be noted that although the results obtained by MC-LMMSE seem to be superior to CKC, it had better be further confirmed by an independent research team.

IPTs can be relatively easily reconstructed from sEMG signals with a low degree of MUAP superposition as long as *d*_*k*_ in Step 4 is similar to *N*_*p*_ (Table 1). However, it will be difficult to satisfactorily reconstruct the IPTs from sEMG signals with a high degree of MUAP superposition in cases where *d*_*k*_ is small. For these scenarios, more iteration steps will be needed to optimize the vector *f* to adequately reconstruct the IPTs (Figures 3(b) and 4).

In order to evaluate the performance of the proposed MC-LMMSE method in experimental sEMG decomposition, the 64 sEMG channels were divided into two independent groups with equal numbers of channels in each. The “two sources” method was employed to compare MUs which were independently decomposed from the two groups of the sEMG signals. Comparison results in Table 2 confirm the high stability, efficiency, and accuracy of the MC-LMMSE method in experimental sEMG decomposition. As a correlation method, the MC-LMMSE method requires a relatively large number of electrodes to achieve good decomposition results; a reduction in the number of electrodes leads to a reduction in the amount of correlation information, which will affect the number of reconstructed IPTs in decomposition results. Consequently, it is necessary to increase the number of recording electrodes under the premise that the amount of information in the sEMG is fully provided if a large number of extracted MUs are desired, particularly in cases of relatively high muscle contraction levels (Table 2).

The major challenges in sEMG decomposition can be summarized as follows [17]: (1) the occurrence of large amounts of superposition between the action potentials from different MUs; (2) the changes in shapes of the different action potentials contained in every MUAP train; (3) high degree of similarity in action potential shapes between different MUAP trains. Those challenges can be overcome to some extent by using the proposed MC-LMMSE method. IPTs can still be reconstructed with high accuracy even if they have a high degree of MUAP superposition (Figures 3(b) and 4). The shapes of the action potentials in MUAP trains may change during the isometric muscle contractions as a result of the changes in conduction velocity (e.g., caused by muscle fatigue) or movement of the electrode, making the decomposition task more challenging. However, even if a large degree of change in MUAP shape occurs quickly, the MC-LMMSE method can still be used to reconstruct the IPTs efficiently and accurately by increasing the iterations in Step 4 or by dividing the signal recordings into short epochs, which could then be considered stationary an absent of shape changes. It is unlikely that the shapes of the action potentials contained in different MUAP trains are similar across all observed channels and, as a result, the correlation between the measurements vectors associated with different MUs can be neglected. Note that both MC-LMMSE and CKC build on the low probability of different MUs to share the exact firing time [18]. MU synchronization does affect the decomposition performance. How much it affects in detail depends on the level of the synchronization and the complexity of the sEMG signal, such as the degree of MUAP waveform superposition, the amount of noise, and so on. In fact, it is extremely difficult to encounter a very high synchronous rate when decomposing real sEMG signals. The formula of probability of synchronization rate was given in (12) of [18]. It can also be seen from the formula that the probability of synchronization is very low. The literature [36] also shows that in the case of very high synchronization rate, it can still be decomposed well by GCKC method. CKC, GCKC, and MC-LMMSE are all based on LMMSE and correlation methods. If GCKC and CKC can do that, the method in this study can also do that.

## 5. Conclusions

In summary, a new MC-LMMSE method was developed for multichannel sEMG decomposition based on the principle that the measurement vectors associated with the firing times of a single MU have a certain degree of similarity. The MC-LMMSE method gradually and iteratively increases the correlation between the optimized vectors and the reconstructed matrix to better decompose complex sEMG signals. The superior performance of the MC-LMMSE method was demonstrated with both simulated and experimental electrode array sEMG signals. The results show that, in each case, the MC-LMMSE method can extract a relatively large number of MUs with strong robustness to noise and excellent accuracy.

## Figures and Tables

**Table 1:** The number of reconstructed IPTs (Nr) (mean ± std. dev.), true positive rate (TPR) (mean ± std. dev.), and misplaced rate (MR) (mean ± std. dev.) for different decomposition methods.

Methods	Parameters	SNR (dB)				
		−10	−5	0	5	10
Measurement matrix autocorrelation	Nr	8.2 ± 1.5	9.6 ± 0.6	9.6 ± 0.6	9.8 ± 0.5	9.8 ± 0.5
	TPR (%)	85.7 ± 1.9	97.6 ± 0.9	99.1 ± 0.3	99.4 ± 0.2	99.4 ± 0.5
	MR(%)	3.98 ± 1.06	2.16 ± 0.67	1.02 ± 0.23	0.98 ± 0.13	0.91 ± 0.06

GCKC	Nr	5.0 ± 0.7	9.0 ± 1.2	10 ± 0	10 ± 0	10 ± 0
	TPR (%)	85.9 ± 4.0	99.5 ± 0.2	99.9 ± 0.1	99.6 ± 0.4	99.9 ± 0.0
	MR(%)	3.59 ± 1.12	1.05 ± 0.33	0.69 ± 0.26	0.66 ± 0.35	0.58 ± 0.17

MC-LMMSE	Nr	10 ± 0	10 ± 0	10 ± 0	10 ± 0	10 ± 0
	TPR (%)	92.8 ± 1.0	99.7 ± 0.0	100 ± 0	100 ± 0	100 ± 0
	MR(%)	2.81 ± 0.85	1.02 ± 0.13	0 ± 0	0 ± 0	0 ± 0

**Table 2:** Parameters (mean ± std. dev.) obtained from all channels and two independent channel groups.

Methods	Contraction force (*N*)	2	4	6	8
GCKC	Number of MUs extracted from all channels	5.7 ± 2.5	8.0 ± 0.0	5.7 ± 4.0	6.7 ± 2.3

MC-LMMSE	Number of MUs extracted from all channels	11.7 ± 4.5	13 ± 1.7	11 ± 3.5	13 ± 1.5
	Number of MUs extracted from channels in Group 1	9.3 ± 4.5	9.0 ± 0.0	7.7 ± 3.5	7.3 ± 1.5
	Number of MUs extracted from channels in Group 2	8.7 ± 3.5	9.0 ± 1.0	7.7 ± 2.1	8.0 ± 1.7
	Number of common MUs extracted from both groups	7.7 ± 4.0	8.7 ± 0.6	6.7 ± 2.5	7.3 ± 1.5
	Percentage of common pulses in common MUs (%)	90 ± 6	92 ± 5	94 ± 4	95 ± 5

## Data Availability

The data used to support the findings of this study are available from the corresponding author upon request.
